# Phantom Eye Syndrome: A Review of the Literature

**DOI:** 10.1155/2014/686493

**Published:** 2014-12-07

**Authors:** Agda M. Andreotti, Marcelo C. Goiato, Eduardo P. Pellizzer, Aldiéris A. Pesqueira, Aimée M. Guiotti, Humberto Gennari-Filho, Daniela M. dos Santos

**Affiliations:** Department of Dental Materials and Prosthodontics, Araçatuba Dental School, São Paulo State University (UNESP), José Bonifácio 1193, 16015-050 Araçatuba, SP, Brazil

## Abstract

The purpose of this literature review was to describe the main features of phantom eye syndrome in relation to their possible causes, symptoms, treatments, and influence of eye amputation on quality of life of anophthalmic patients. For this, a bibliographical research was performed in Pubmed database using the following terms: “eye amputation,” “eye trauma,” “phantom eye syndrome,” “phantom pain,” and “quality of life,” associated or not. Thirteen studies were selected, besides some relevant references contained in the selected manuscripts and other studies hallowed in the literature. Thus, 56 articles were included in this review. The phantom eye syndrome is defined as any sensation reported by the patient with anophthalmia, originated anophthalmic cavity. In phantom eye syndrome, at least one of these three symptoms has to be present: phantom vision, phantom pain, and phantom sensations. This syndrome has a direct influence on the quality of life of the patients, and psychological support is recommended before and after the amputation of the eyeball as well as aid in the treatment of the syndrome. Therefore, it is suggested that, for more effective treatment of phantom eye syndrome, drug therapy should be associated with psychological approach.

## 1. Introduction

The loss of an eye directly influences the quality of life of the anophthalmic individuals and may trigger several social, familiar, and psychic problems, resulting in devastating emotional effects on the patient, such as fears of meeting new people, difficulties of establishing emotional ties and organizing their lives in the face of new circumstances, and feelings of insecurity and fear of being socially outcast [1].

Three types of surgery are applied when performing eye amputation: enucleation is the removal of the globe from the orbit, involving the separation of all connections between the globe and the patient including the sclera [2], evisceration is a procedure in which the intraocular contents are removed while the sclera, Tenon's capsule, conjunctiva, and optic nerve are preserved [3], and exenteration is a radical procedure consisting of removal of the orbital contents, including orbital fat, conjunctival sac, globe, and part or all of the eyelids [2, 4].

The great majority of eye amputations are undertaken to treat potentially life threatening malignancies [2–6] or implacably progressive conditions unresponsive to other treatments [4]. The eye amputation may also aid in attenuation of severe deformity, pain or less often aid as treatment for nonmalignant disease [4, 7]. The three most common indications for enucleation are intraocular malignancy, a blind painful eye [3, 5], prevention of sympathetic ophthalmia, phthisis [2, 5], microphthalmia in a child to enhance bony orbital development, improvement of cosmesis [2], trauma, and miscellaneous among others [5].

These surgeries may bring up some complications grouped according to time of their appearance: surgical complications occur at the moment of the surgery; postoperative ones occur within the first days to months after surgery; and late complications normally occur months to years after surgery and among them, is the phantom eye syndrome [2, 8–10].

Phantom eye syndrome is classified as painless and painful sensations referred to the amputated eye and clearly distinguishable from both cicatrix pain and any other sensory disturbances in or around the cicatrix [11], and it is always associated with phantom vision, phantom pain, and phantom sensations [8, 10, 12, 13]. After limb amputation, more than 90% of patients experience phantom phenomena [12, 14]. Studies report that 51% of people who have lost an eye can suffer from phantom eye syndrome [8]. Others claim that 46% of patients with eye amputation present at least one of the typical symptoms of phantom eye syndrome (visual hallucination, phantom pain, or phantom sensation) [12].

Most reports of phantom sensations and phantom pain deal with phantom limbs. Very little is written about phantom eye syndrome, possibly because removal of the ocular bulb is mostly performed to treat orbital area cancer, a condition with very low prevalence [11]. Given that this is a relatively new area and despite being a high prevalence phenomenon, this syndrome is scarcely discussed in published literature [10]. Thus, the aim of this study was, by reviewing the current literature, reporting the main features of the phantom eye syndrome in relation to their possible causes, symptoms, treatments, and influence of eye amputation in quality of life of anophthalmic patients.

## 2. Material and Methods

Literature search was performed in Pubmed database using the following terms and associations between them: “eye amputation,” “eye amputation AND phantom pain,” “eye amputation AND phantom eye syndrome,” “eye amputation AND quality of life,” “eye trauma AND phantom pain,” “eye trauma AND phantom eye syndrome,” “eye trauma AND quality of life,” “anophthalmia” AND “quality of life,” and “phantom eye syndrome.” The articles were selected according to the inclusion and exclusion criteria (Table 1).

Moreover, as few articles about the phantom eye syndrome were found, some articles that contain information about phantom pain in eye and in other limbs, than the eye, have also been selected, since the principle that leads to some phantom pain is the same for all organs.

Despite being published in another period than the determined in inclusion and exclusion criteria, some studies about phantom eye syndrome were also included, because they are hallowed in literature and have great importance in the subject of this study.

## 3. Results

By using the keywords mentioned, 167 articles were found in Pubmed database, 13 of which were selected to compose this review, according to the inclusion and exclusion criteria. We also included studies contained in the references of selected articles that are relevant to the purpose of this review but were not found in the search using the keywords proposed. Some studies about phantom eye syndrome were also included, since they are hallowed in literature and have great importance in the subject of this study.

## 4. Review and Discussion

The phantom eye syndrome is associated with visual hallucinations, phantom pain, and phantom sensations.

### 4.1. Visual Hallucinations

Visual hallucinations are illusory perceptions in the removed eye owing a sense of reality but occurring without external stimulation of the sensory organ [12]. People, animals, buildings, and scenery are most often reported. The vision is described as well-defined, organized, and clear and may represent a release of the visual association cortex from vision; however, its mechanism is poorly understood [15].

Visual hallucinations may be elementary or complex. Elementary visual hallucinations include simple visual phenomena lacking meaning and form, while complex visual hallucinations consist of formed contours, objects, scenes, or persons, sometimes related to past experiences of the subject [10, 12, 16, 17]. The elementary and complex hallucinations might be thought of as “true hallucinations”; that is, something which is not present in the external world is perceived [17].

Sörös et al., 2003 [12], stated that most patients with visual hallucinations report basic perceptions and that complex perceptions are less frequent, which corroborates Rasmussen et al., 2009, who reported that, from around 37 anophthalmic patients with visual hallucinations, 36% exhibited elementary visual hallucinations, and only 1% had complex visual hallucinations [10]. This is also related by Wilkinson [17], who stated that simple hallucinations form the majority in any study in which all types of hallucinations are evaluated, despite the fact that emphasis on complex hallucinations in many studies gives a different impression.

Probably because of the variety of levels and etiologies, the literature shows no consistent suggestions of either the factors triggering such hallucinations, the conditions necessary and sufficient to sustain them, or the effective methods of their suppression [17]. These hallucinations typically occur independently of any triggering factors or exercise of volition in the genesis of the image. In some individuals, however, they may be triggered by a wide variety of stimuli such as conditions of general sensory reduction, fatigue, stress, and low levels of illumination or even by bright light [10, 12, 16, 17].

According to Kolmel [18], onset is typically within a few days of the event causing the anophthalmia and frequency generally decreases over time. Once manifested, images last for periods varying from seconds through minutes to hours [15]. Santhouse et al. [19] reported that, from 123 patients with hallucinations, 68% related that hallucinations occurred at least daily, with 23% hallucinating at least hourly or constantly. There was a tendency for hallucinations to last for minutes, rather than seconds or hours. These hallucinations can subsequently disappear, either spontaneously or in response to actions such as closing the eyes [16]. There are reports that these hallucinations may surcease in a period between a few weeks and 6 months after the eye amputation [10, 16].

Phantom vision is a real and detailed phenomenon that is a concern to patients [15]. Historically, hallucinatory experiences have been deemed to signify mental instability; patients are therefore often reluctant to admit to their hallucinatory experiences [16]. So, they need to be reassured that these sensations are benign and do not signify psychiatric or psychological illness [15].

### 4.2. Phantom Pain

Phantom pain is a form of neuropathic pain [20], which may be caused by a lesion or disease of the somatosensory system [21], or may be related to damage of central or peripheral neurons [22]. It is defined as a feeling of pain in the limb that is no longer present [8, 12, 13, 23, 24], being considered an aftereffect of amputation [24] and affecting a large proportion of amputees, with an incidence of 50–85% [20, 22, 25–28].

The pain may be related to a specific position or movement of the phantom limb and can be caused or exacerbated by a number of physical factors, such as changes in climate or pressure on the remaining limb [22]. Some studies suggest a relationship between phantom limb pain and etiology of amputation and preamputation pain [23, 24, 29, 30]. Jensen et al., 1985 [31], report that the presence of preamputation pain may increase the risk of phantom pain after amputation and that longer duration of preamputation pain is also a risk factor for chronic phantom pain. These findings are in accordance with Weiss and Lindell, 1996 [32], who state that more severe pain etiologies have been associated with more severe phantom pain after amputation, compared with less severe pain etiologies. Sörös et al., 2003 [12], also demonstrated a significant association between painful and nonpainful phantom experiences and preoperative pain in the symptomatic eye. However, Nicolodi et al., 1997 [11], demonstrated no connection with preamputation pain and phantom pain.

Moreover, there is increasing evidence that phantom pain results from improperly stored or chronically activated pain memories. Case studies have reported examples of pain “memories,” in which the painful phantom sensations resemble a type of pain experienced before amputation [26, 27, 29, 33, 34].

Phantom pain is also recognized to be an interaction of physical and psychological factors. Importantly, emotion can be a central factor in the production and maintenance of pain [26]. Patients who received less support before amputation tend to report greater pain in the phantom limb [35].

Studies relate that the onset of pain is early. Several studies have shown that 75% of patients develop pain within the first few days after amputation [36–38]. However, phantom pain may be delayed for months or years. Most patients with phantom pain have intermittent pain, with intervals that range from 1 day to several weeks. Even intervals of over a year have been reported. The pain often presents itself in the form of attacks that vary in duration from a few seconds to minutes or hours [23].

According to Gerding et al., 2003 [30], phantom eye pain is present in nearly 1/4 of patients after enucleation. As said previously, Rasmussen et al. in 2011 [28] conducted a study with 173 anophthalmic patients and reported that 23% of these patients had phantom pain, which is similar to Sörös et al., 2003 [12], who reported that, in a population of 123, the prevalence of phantom pain was 26%. But literature is still sparse regarding studies that associate phantom pain with eye amputation.

The pain coming from an anophthalmic cavity is originated from a dysfunctional cavity, indicating that there is a structural or pathological cause for pain, such as, conjunctival cysts, migration of the implant, lacrimal insufficiency, infectious or inflammatory conditions, tumors, hematoma, residual silicone, brain and diseases, compression or irritation of the trigeminal nerve, psychological causes, and the need for modification of prostheses [28, 39]. As there are no studies that describe the quality of the phantom eye pain, the comparison is made with studies about phantom pain in amputated limbs, and in those cases, the phantom limb pain is often described as shooting, stabbing, penetration, cramps, pinch, burning, crushing, shocking, sticking, and cramping [23, 24, 28, 40].

Most patients with phantom pain present intermittent pain, with intervals varying from one day to several weeks. Even intervals over a year have been reported. The pain often presents itself in the form of attacks that vary in duration from a few seconds to a few minutes or hours [23, 24]. Phantom pain after eye amputation is relatively common and patients should be informed about potential complications before surgery.

### 4.3. Phantom Sensations

Phantom sensation is any sensation (paresthesia, dysesthesia, and hyperpathia) of the missing limb except pain [41], which has been accepted as normal sequel to amputation [40]. It usually manifests as kinetic, kinesthetic, or exteroceptive perceptions [23].

The phantom sensation is experienced by almost everyone who undergoes limb amputation, but it is rarely a clinical problem [24]. Immediately after amputation, phantom limb generally resembles the preamputated limb in shape, length, and volume. The sensations can be very vivid and usually include sensations of posture, movement, heat, and tingling. Nonpainful sensations are not observed immediately after surgery. Their incidence is generally highest 2–12 months postoperatively. Over time the phantom sensation may disappear [11, 23, 24].

The eye not only has rich somatosensory innervations, predisposing for the development of phantom sensations, but also is the most important sensory organ with the largest cortical representation in humans [12]. However, the incidence of phantom sensations in anophthalmic cavity is low and manifests as itching around the eyes, feeling of nonexisting eyelids, and sensation of opening and seeing with both eyes [8].

Initial information should try to reassure the patient that the sensations and pains that they experience are normal, are not imagined, and do not necessarily signify the occurrence of any complicating conditions or reemergence of a past disease [40].

### 4.4. Treatment

Prevention of phantom eye syndrome is important and whether the benefit expected with this enucleation overcomes the drawbacks resulting from the syndrome must be assessed [8].

The management of phantom phenomena is challenging because the response to most drugs remains unpredictable despite attempts to develop a more rational therapeutic approach [21].

Nevertheless, pharmacologic therapy represents the most noninvasive option for treatment of phantom pain. Optimum treatment of this challenging condition may involve combinations of pharmacologic and nonpharmacologic treatments [20]. Surgery, drug therapy, and psychological approach are suggested as treatments for phantom limb syndrome [27]; however, when it comes to phantom eye syndrome, the literature is limited and does not point out a treatment protocol.

Some drugs are associated with the treatment of phantom phenomena, such as antidepressants [22, 23, 25, 28, 42, 43], anticonvulsants [20, 22–24, 28, 42, 44, 45], sodium channel blockers [11, 12, 17, 25, 31], N-methyl-D-aspartate receptor antagonists [21–24, 28, 42, 44, 46–48], and opioids [21–24, 28, 42, 43, 45, 49], but other medications are also reported (Table 2) [22–24, 28, 42, 50–52].

Despite such suggestions, efforts are needed in order to recover the patient with this syndrome. This recovering implies improved quality of life and reintegration into social life.

Anophthalmic patients have a lower quality of life in comparison with the general population [53], given the central role of the eyes in communication and physical attractiveness [54]. Eye amputation can result in emotional devastating problems such as insecurity, rejection, inferiority complexes, and fear of social marginalization.

Some studies relate that patients who underwent surgical removal of the eye reported that depression was overcome after installation of ocular prosthesis [55]. The main function of this prosthesis is to keep the socket continuing to perform many of the functions of a normal eye socket, such as blinking, winking, and even shedding tears, since the lids and tear glands are still in working order [56]. Therefore, besides restoring aesthetics and protecting the damaged area, the prosthesis can promote psychological restructuring in a certain degree, which may lead to resolution of some disorders; however for the great psychic trauma, individualized psychological support is essential [57].

Ahn et al., in 2010, highlighted the need for attention directed towards improving the quality of life and reducing anxiety and depression in patients affected, requiring, in addition to medical support, psychological support to deal with this condition. The mental health of anophthalmic patients should be measured initially and checked regularly to assure that there is no development of anxiety or depression disorders. If additional professional help is necessary, patients should be referred to psychiatrists for a deeper consultation and appropriate treatment strategies [1].

## 5. Final Considerations

From the literature review it can be concluded the following.

- The phantom eye syndrome has as etiology the evisceration, enucleation, or exenteration of one or both eyes, affecting sensory and motor nerves.
- The symptoms often related to this syndrome are visual hallucinations, phantom pain, and phantom sensations.
- This syndrome has a great impact on the quality of life of patients, and psychological support before and after the amputation of the eyeball as well as a complementary treatment of the syndrome is recommended.
- Drug therapy is greatly associated with this type of syndrome, although not providing satisfactory results in all cases.
- A psychological approach associated with drug therapy should be indicated as treatment for patients with phantom eye syndrome.

## Figures and Tables

**Table 1:** Inclusion and exclusion criteria for selection of articles.

Inclusion criteria	Exclusion criteria
Articles in English	Articles in other languages

Articles published between 2000 and 2012	Articles not published from 2000 to 2012

Articles that report major cause of eye loss and indications for surgical removal	Articles that do not cite the phantom eye syndrome as a result of eye loss

Articles that report the phantom eye syndrome as a result of eye loss	Articles that do not contain information about the phantom eye syndrome, as causes, characteristics, symptoms, and treatment

Articles that report at least one of the following: causes, characteristics, symptoms, and treatment options for phantom eye syndrome	—

Articles that report the influence of eye loss in quality of life	—

**Table 2:** Drugs used in the treatment of phantom phenomena.

Pharmacological class	Drugs
Antidepressants	Amitriptyline^x^
	Tricyclics^x^
	Duloxetine
	Milnacipran

Anticonvulsants	Gabapentin^x^
	Pregabalin^x^
	Carbamazepine^x^
	Oxcarbazepine^x^

Sodium channel blockers	Bupivacaine^x^
	Lidocaine
	Mexiletine

N-Methyl-D-aspartate receptor antagonists	Ketamine^x^
	Memantine^x^

Opioids	Morphine^x^
	Methadone
	Buprenorphine^x^
	Tramadol^x^

Others	Calcitonin^x^
	Benzodiazepine
	Acetominophen^x^
	Nonsteroidal anti-inflammatory^x^
	Beta blockers^x^
	Muscle relaxants^x^
	Corticosteroids^x^
	Neuroleptics^x^
	Barbiturics^x^

^x^Drugs with reports of improvements in the clinical aspect of the phantom phenomena.
